# Primary and Secondary Bystander Effects of Proton Microbeam Irradiation on Human Lung Cancer Cells under Hypoxic Conditions

**DOI:** 10.3390/biology12121485

**Published:** 2023-12-03

**Authors:** Narongchai Autsavapromporn, Alisa Kobayashi, Cuihua Liu, Aphidet Duangya, Masakazu Oikawa, Tengku Ahbrizal Tengku Ahmad, Teruaki Konishi

**Affiliations:** 1 Division of Radiation Oncology, Department of Radiology, Faculty of Medicine, Chiang Mai University, Chiang Mai 50200, Thailand; aphidet.d@cmu.ac.th; 2 Single Cell Radiation Biology Team, National Institutes for Quantum Science and Technology, Chiba 263-8555, Japan; 3 Molecular and Cellular Radiation Biology Group, National Institutes for Quantum Science and Technology, Chiba 263-8555, Japan; liu.cuihua@qst.go.jp; 4 Electrostatic Accelerator Operation Section, National Institutes for Quantum Science and Technology, Chiba 263-8555, Japan; oikawa.masakazu@qst.go.jp; 5 Radiation Safety and Health Division, Malaysian Nuclear Agency, Bangi, Kajang 43000, Selangor, Malaysia; ahbrizal@nm.gov.my

**Keywords:** proton microbeam, primary and secondary bystander effect, hypoxia, gap–junction, NO

## Abstract

**Simple Summary:**

The SPICE-QST proton microbeams (3.4 MeV proton, LET~11.7 keV/µm) are powerful tools to investigate the mechanism(s) underlying radiation-induced bystander effects (RIBE) and radiation-induced secondary bystander effect (RISBE) in hypoxic cells. The purpose of this study was to elucidate the RIBE and RISBE in hypoxic lung cancer cells following high-LET proton irradiation. Specifically, the role of intercellular communication through gap–junction intercellular communication (GJIC) and/or soluble factors from irradiated to nonirradiated cells, such as nitric oxide (NO), is the major mechanism responsible for the observed effects. Our results indicate that the propagation of RIBE and RISBE in hypoxic cancer cells depends on the radiation dose and oxygen status. These results also highlight the key role of intercellular communication via GJIC in the modulation of stressful effects in primary and secondary bystander cancer cells under hypoxic conditions, which may contribute to improving the treatment outcomes.

**Abstract:**

Tumor hypoxia is the most common feature of radioresistance to the radiotherapy (RT) of lung cancer and results in poor clinical outcomes. High-linear energy transfer (LET) radiation is a novel RT technique to overcome this problem. However, a limited number of studies have been elucidated on the underlying mechanism(s) of RIBE and RISBE in cancer cells exposed to high-LET radiation under hypoxia. Here, we developed a new method to investigate the RIBE and RISBE under hypoxia using the SPICE-QST proton microbeams and a layered tissue co-culture system. Normal lung fibroblast (WI-38) and lung cancer (A549) cells were exposed in the range of 06 Gy of proton microbeams, wherein only ~0.04–0.15% of the cells were traversed by protons. Subsequently, primary bystander A549 cells were co-cultured with secondary bystander A549 cells in the presence or absence of a GJIC and NO inhibitor using co-culture systems. Studies show that there are differences in RIBE in A549 and WI-38 primary bystander cells under normoxia and hypoxia. Interestingly, treatment with a GJIC inhibitor showed an increase in the toxicity of primary bystander WI-38 cells but a decrease in A549 cells under hypoxia. Our results also show the induction of RISBE in secondary bystander A549 cells under hypoxia, where GJIC and NO inhibitors reduced the stressful effects on secondary bystander A549 cells. Together, these preliminary results, for the first time, represented the involvement of intercellular communications through GJIC in propagation of RIBE and RISBE in hypoxic cancer cells.

## 1. Introduction

Radiotherapy (RT) is an important therapeutic modality used for lung cancer treatment. RT, particularly high-linear energy transfer (LET) radiation, has the advantage of Bragg peak area to increase dose deposition in a tumor, thereby having less impact on surrounding healthy normal tissues or organs. Furthermore, high-LET radiation is considered the most advanced and novel technology in RT [1]. For example, proton microbeams (3.4 MeV, High LET~11.7 keV/µm) using the single-particle irradiation system to cell (SPICE) at the National Institute for Quantum Science and Technology (QST) is the only proton microbeam facility in Japan and Asia where single cell irradiation can be performed by many in vitro and in vivo studies [2,3,4]. The SPICE-QST proton microbeam is a valuable and powerful tool for studying radiation-induced bystander effects (RIBE). RIBE occurs when nonirradiated bystander cells receive intercellular signaling molecules produced by irradiated cells. This leads to biological damage in bystander cells [5,6]. Further, it is important to note that primary bystander cells can also transmit a variety of signaling molecules to their neighbors, which is defined as a radiation-induced secondary bystander effect (RISBE) [7].

Importantly, RIBE and RIBSE may be either harmful or beneficial during RT and may cause a decrease in the therapeutic ratio in lung cancer treatment [7,8]. However, the mechanisms of RIBE and RIBSE are complex and not clearly defined. Several studies have been conducted to extensively identify mechanisms mediating RIBE and RIBSE, such as oxidative metabolism, inflammatory responses, secreted diffusible factors (such as nitric oxide (NO), cytokines, and chemokines) from irradiated cells or primary bystander cells, extracellular vesicles (such as exosomes) from irradiated cells to nonirradiated bystander cells, and direct intercellular communications between cells via gap–junction intercellular communication (GJIC) [8,9,10,11,12]. Recently, we have demonstrated the role of GJIC and secreted diffusible factors in mediating the RIBE and RIBSE in normal and cancer cells exposed to proton microbeam under normoxia, which suggests that RIBE and RIBSE may potentially impact on RT by increasing the effectiveness of killing cancer cell [7]. 

It has been noted that hypoxia (with low O_2_ < 2%) is considered one of the major causes of radioresistance and failure in lung cancer treatment during RT [13]. This situation is caused by a low level of dissolved oxygen or a lack of oxygen concentration in cells. This leads to a reduction in ROS generation, thereby decreasing tumor cell killing following RT, which is particularly true for low-LET radiation. This contrasts with high-LET radiation, which is less dependent on ROS generation (hypoxic conditions) [14,15]. There are few studies on RIBE in hypoxic cancer cells exposed to high-LET radiation. In a previous study, we found that there are different roles of RIBE in cancer and normal cells exposed to high-LET radiation under hypoxic conditions. These results suggest that GJIC can play a critical role in RIBE [3]. However, studies on the role of RISBE on hypoxic lung cancer cells using high-LET radiation are relatively limited. Considering the importance of hypoxia-induced bystander effects and high-LET radiation, the aim of this study is to investigate RIBE and RIBSE on hypoxic lung cancer cells exposed to high-LET microbeams with SPICE-QST protons.

## 2. Materials and Methods

### 2.1. Cell Culture

We used two types of cell lines in this study: normal lung fibroblast (WI-38) and human non-small-cell lung adenocarcinoma (A549) cells. Both cell lines were obtained from the RIKEN Bioresource Center (Ibaraki, Japan). The cells were grown in culture flasks using D-MEM (Wako Pure Chemical Industries Ltd., Osaka, Japan) supplemented with 10% fetal bovine serum, 100 U/mL penicillin, and 100 µg/mL streptomycin (ThermoFisher Scientific, Tokyo, Japan) and maintained in 5% CO_2_ at 37 °C. For hypoxic treatment, both cells were incubated at 37 °C under 1% O_2_, 5% CO_2_, and 94% N_2_ conditions in a humidified multigas incubator (MCO-5M-PJ, Panasonic Corporation, Osaka, Japan).

### 2.2. Sample Preparation and Proton Microbeam Irradiation 

**Figure 1:**
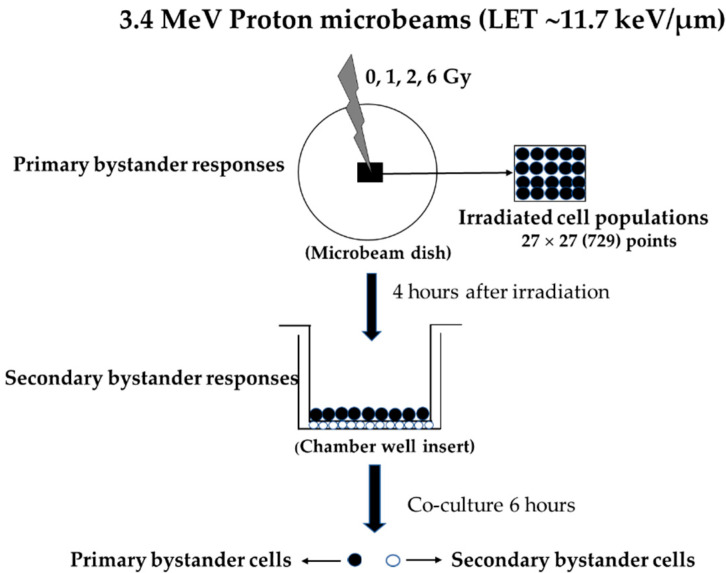
Scheme representation of co-culture methodology to investigate the RIBE and RISBE.

The proton irradiation was performed using a Single-Particle Irradiation System to Cells (SPICE) located at the National Institutes for Quantum and Radiological Science and Technology (QST) in Chiba, Japan. The facility is equipped with a Tandetron 1.7-MV accelerator manufactured by Europa High Voltage Engineering B.V. (HVEE, Amersfoort, The Netherlands), which supplies protons and helium ions [2]. In a vertical line, 3.4 MeV protons can be delivered in a beam diameter of 2 µm (target cell distance is before the Bragg peak: LET~11.7 keV/µm at the cell entrance). The beam characteristics, dosimetry, and biological irradiation procedures of SPICE-QST proton microbeam have been described in detail elsewhere [2]. Briefly, approximately 1 × 10^5^ cells of WI-38 or A549 cells were seeded in a specially designed microbeam cell culture dish with 6 µm thick polypropylene film (Chemplex Industries Inc., Plam City, FL, USA) underneath. Cells were cultured for 2 days under normoxia (21% O_2_) or hypoxia (1% O_2_) to ensure that both cell cultures were fully confluent (~94% in G_1_/G_0_ phase). This is to allow direct intercellular communication through GJIC between cells. Prior to irradiation, culture medium was removed, and the microbeam dish was covered with 6 µm polypropylene film to keep cells hydrated during microbeam irradiation process. The direction of beam was undertaken from bottom to top of microbeam dish. The cytoplasm or cell nucleus in the center of the dish (A total of 27 × 27 = 729 points) was irradiated under normal oxygen conditions at room temperature, as shown in Figure 1. Within each point, 45, 90, and 270 protons were delivered, and the absorbed dose was estimated to be 1, 2, and 6 Gy, respectively. Microbeam irradiation process was completed in less than 10 min. The fraction of WI-38 and A549 cells by a primary proton in the exposed population was estimated to be 0.04–0.15%. Thus, most of the cells in the dishes were represented as bystander cells. Immediately after microbeam irradiation, D-MEM medium was added to the cell dishes. In this step, the polypropylene film was removed and cultured for another 4 h at 37 °C under hypoxia or normoxia. Sham-irradiated cells that were handled similarly to the bystander cells served as controls.

### 2.3. Co-Culture System to Elucidate Radiation-Induced Secondary Bystander Effect

To investigate radiation-induced secondary bystander effect (RISBE), a layered tissue co-culture system was used (Figure 1). Briefly, 2 days before irradiation, cells that would become secondary bystander A549 cells (~1 × 10^5^ cell) were trypsinized and seeded into inverted transwell culture inserts with a pore size of 3 µm in order to allow intercellular communication between cell populations at 37 °C under hypoxia. After proton microbeam irradiation, primary bystander A549 cells (~1 × 10^5^ cell) were seeded at the top of co-culture inserts dish with confluent secondary bystander A549 cells growing at the bottom side of the insert. After co-culturing for additional 6 h, cells were collected and assayed for further analyses.

### 2.4. Clonogenic Survival Assay

A549 and WI-38 cells were trypsinized, counted, and seeded into P100 petri dish under hypoxic or normoxic conditions for 8 and 12 days, respectively. The culture medium was then discarded, and cultures were rinsed with phosphate-buffered saline (PBS). Colonies were fixed with 99.5% ethanol, stained with 0.2% crystal violet solution, and left to air-dry. Colonies containing more than 50 cells were scored using light microscopy. Cell counts were normalized to control cells (nonirradiated). Plating efficiency (PE) was calculated using the following Equation (1):

PE = number of colonies count/number of cells seeds × 100%(1)

### 2.5. Micronucleus Formation Assay

Micronucleus (MN) formation was performed to determine DNA damage (genotoxicity) in primary and secondary bystander cells using cytokinesis-block technique [16]. Briefly, approximately 5 × 10^4^ cells were counted and seeded in a 4-well chamber flask containing 2 µg/mL cytochalasin B (Sigma, St. Louis, MO, USA) in D-MEM culture media. A549 and WI-38 cells were incubated under normoxia or hypoxia for 48 and 72 h, respectively, to obtain binucleated cells. By the end of the incubation period, cells were washed with PBS and then fixed with 99.5% ethanol. Cells were then stained with 1 µg/mL of Hoechst 33342 solution and observed under a fluorescence microscope. A minimum of 1000 binucleated cells for each sample was scored, and only micronuclei in binucleated cells were selected.

### 2.6. Chromosome Aberration Assay

Cells were seeded into T25 flasks and incubated under hypoxia for 24 h. Cells were then treated with Colcemid (final concentration of 0.05 mg/mL) and incubated for another 5–6 h. Subsequently, cells were harvested and resuspended with 75 mM potassium chloride (KCI) at 37 °C. The cells were fixed with 5 mL of Carnoy’s fixative solution (methanol/acetic acid, 3:1) and washed three times with Carnoy’s fixative solution. The cell suspension was dropped onto ethanol-cleaned slides and left to air-dry. Slides were stained with 2% Giemsa solution. Slides were then rinsed with running tap water, air-dried, and mounted with cover slip. Approximately 500–1000 metaphases for each sample were analyzed for dicentric chromosomes under microscope.

### 2.7. Inhibition of GJIC

Prior to irradiation, cells were treated with 18-α-glycyrrhetinic acid (AGA, a specific gap–junction inhibitor of GJIC) at 37 °C for 30 min (50 µM, non-toxic concentration). Immediately after irradiation, D-MEM medium supplemented with AGA was added and incubated for another 4–6 h at 37 °C under normoxia or hypoxia for further analysis.

### 2.8. Nitric Oxide (NO) Scavenger

Carboxy-PTIO (c-PTIO) [2-(4-carboxyphenyl)-4,4,5,5,-tertranmethulimidazoline-1-oxyl-3-oxide)] was used to measure the NO scavenger. c-PTIO was dissolved in PBS. Just before co-culturing with primary bystander cells, secondary bystander cells were treated with 20 µM c-PTIO (non-toxic concentration) at 37 °C for 30 min under hypoxia. 

### 2.9. Statistical Analysis

All data are presented as mean ± SE using Sigma Plot 10 software V.10 of at least three independent experiments on different experimental days. The Poisson statistics and Pearson’s chi-square test were used to compare independent samples, and a *p* value of <0.05 was regarded to be statistically significant between comparative groups.

## 3. Results

### 3.1. RIBE in Primary Bystander Normal and Cancer Cells Exposed to Proton Microbeam under Hypoxic and Normoxic Conditions

**Figure 2:**
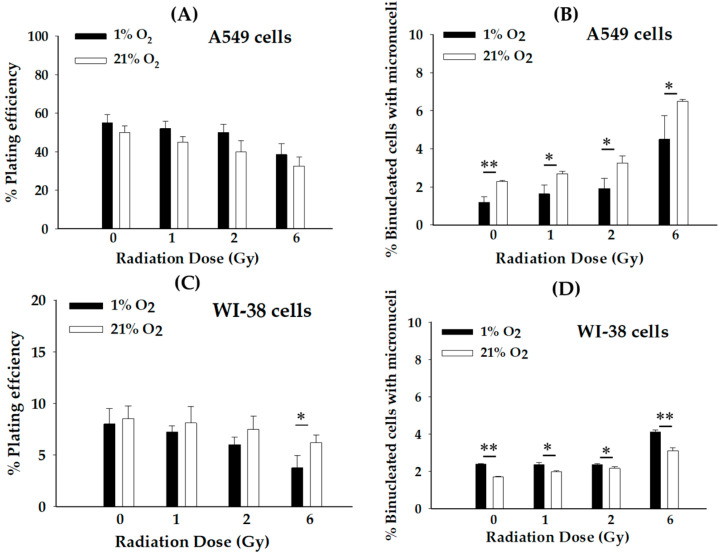
RIBE in primary bystander cancer and normal cells exposed to proton microbeam under hypoxic or normoxic conditions. (**A**) Plating efficiency and (**B**) micronucleus formation of bystander cancer A549 cells. (**C**) Plating efficiency and (**D**) micronucleus formation of bystander normal WI-38 cells. The radiation dose represented in the figure indicates the dose absorbed by proton-irradiated cells. (*n* = 4, * *p* < 0.05 and ** *p* < 0.01).

To elucidate the contribution of RIBE on primary bystander cells under hypoxia and normoxia, we designed an irradiation model with the SPICE-QST proton microbeams (Figure 1). As expected, the result in Figure 2A shows that the plating efficiency (PE) in hypoxic-treated bystander A549 cells was higher than detected under normoxia at all doses tested when compared to control groups. Consistent with the PE results, the frequency of micronucleus (MN) formation in hypoxic-treated bystander A549 cells was significantly (* *p* < 0.05 and ** *p* < 0.01) decreased compared to cells under normoxic conditions (Figure 2B). These results indicate that the hypoxic condition induces a radioresistance characteristic in A549 bystander cells. In contrast, a decrease in PE was observed in bystander hypoxic WI-38 cells compared to normoxic cells at all dose levels (Figure 2C). Consistent with the above findings, the frequency of MN in hypoxic-treated bystander WI-38 cells was significantly (* *p* < 0.05 and ** *p* < 0.01) increased compared to cells under normoxic conditions (Figure 2D). Based on these findings, the RIBE under hypoxic conditions depends on cell type and radiation dose.

### 3.2. Role of GJIC in Propagation of Stressful or Protective Effects in Primary Bystander Normal and Cancer Cells following Proton Microbeam Irradiation under Hypoxic and Normoxic Conditions 

**Figure 3:**
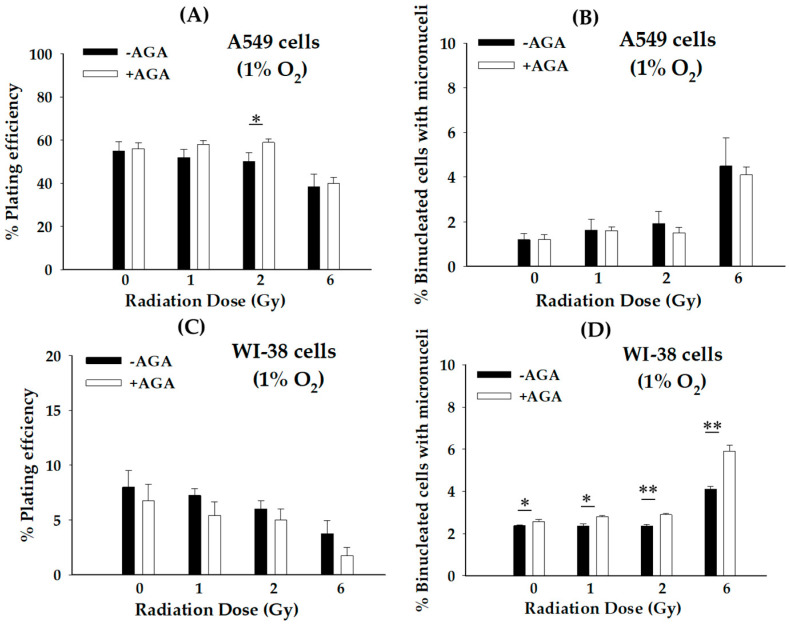
Role of intercellular communication via GJIC in primary bystander A549 and WI-38 cells exposed to proton microbeam under hypoxic conditions in the presence or absence of gap–junction inhibitor (AGA). (**A**) Plating efficiency and (**B**) micronucleus formation of hypoxic-treated bystander A549 cells. (**C**) Plating efficiency and (**D**) micronucleus formation of hypoxic-treated bystander WI-38 cells. The radiation dose represented in the figure indicates the dose absorbed by proton-irradiated cells. (*n* = 4, * *p* < 0.05 and ** *p* < 0.01).

Next, to gain insight into the mechanism(s) underlying radiation toxicity on hypoxic-treated bystander WI-38 cells after proton microbeam irradiation, we investigated whether GJIC is involved in RIBE. It is interesting to note that the D-MEM culture medium was discarded during irradiation. Therefore, the role of secreted diffusible factors from irradiated cells to primary bystander cells is expected to be negligible in these experiments. The results of the study show that the inhibition of GJIC by AGA in hypoxic-treated bystander WI-38 cells decreased the PE (Figure 3C) but significantly (* *p* < 0.05 and ** *p* < 0.01) increased MN (Figure 3D) at all doses compared to their control. These findings were not observed in hypoxic-treated bystander A549 cells. In contrast, the AGA inhibitor increased the PE (Figure 3A) but decreased MN (Figure 3B) for all doses compared to the untreated control. These results are consistent with our previous data showing that different cells have different underlying mechanisms in GJIC characterization [3]. Thus, the involvement of GJIC showed an increased lethal effect in hypoxic-treated bystander cancer cells, whereas it was decreased in hypoxic-treated bystander normal cells with gap–junction closure.

### 3.3. RISBE in Secondary Bystander Cancer Cells Exposed to Proton Microbeam Irradiation under Hypoxic Conditions

**Figure 4:**
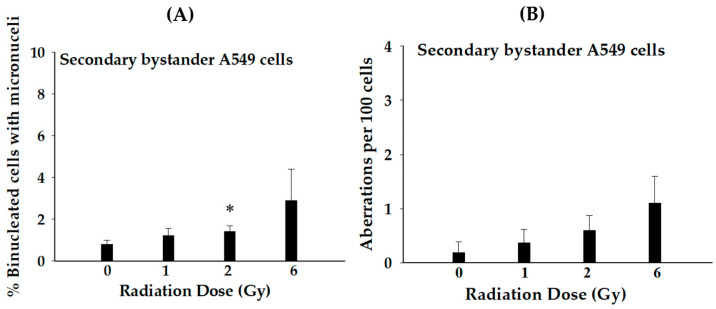
RISBE in the secondary bystander cancer cells exposed to proton microbeam under hypoxia. (**A**) Micronucleus formation and (**B**) chromosome aberration in secondary bystander A549 cells co-cultured with primary bystander A549 cells. The radiation dose represented in the figure indicates the dose absorbed by proton-irradiated cells. (*n* = 3, * *p* < 0.05).

We continued the investigation by identifying the role of RISBE on truly secondary bystander A549 cells co-cultured with primary bystander A549 cells using a layered tissue co-culture system (Figure 1). As indicated in Figure 4A,B, the frequency of MN formation and chromosome aberration (CA) in secondary bystander A549 cells was higher than their respective controls at all doses tested. Together, these data clearly indicate that the induction of RISBE in secondary bystander A549 cells under hypoxia and RISBE are radiation dose-dependent.

### 3.4. Roles of GJIC and Secreted Diffusible Factors in Propagation of Stressful Effect in Secondary Bystander Cancer Cells Exposed to Proton Microbeam Irradiation under Hypoxic Conditions

**Figure 5:**
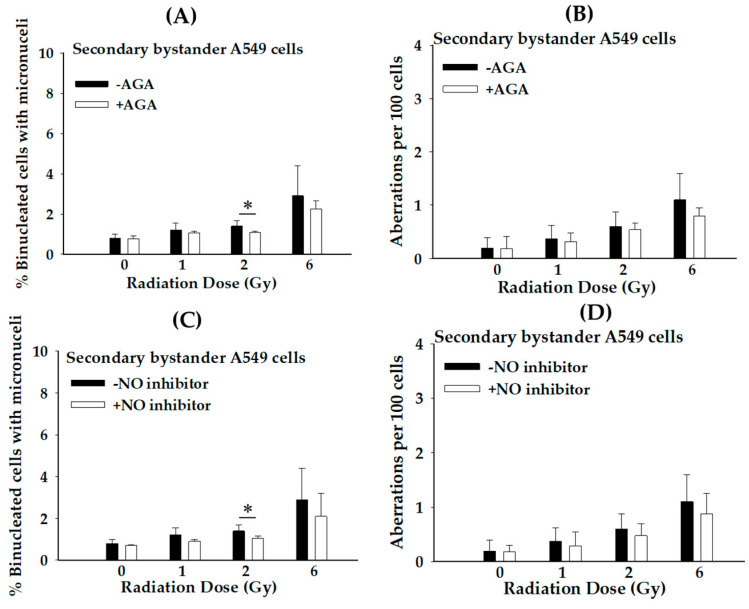
Roles of GJIC and secreted diffusible factors in propagation of stressful effects in the secondary bystander A549 cells under hypoxic conditions after proton microbeam irradiation. (**A**) Micronucleus formation and (**B**) chromosome aberration of secondary bystander A549 cells in the presence or absence of gap–junction inhibitor (AGA); (**C**) micronucleus formation and (**D**) chromosome aberration of secondary bystander A549 cells in the presence or absence of NO inhibitor (c-PTIO). The radiation dose represented in the figure indicates the dose absorbed by proton-irradiated cells. (*n* = 3 and * *p* < 0.05).

In the next study, we investigated the involvement of GJIC and secreted diffusible factors related to the propagation of lethal effects on truly secondary bystander A549 cells under hypoxic conditions following exposure to proton microbeams. Surprisingly, Figure 5A,B show that treatment with a GJIC inhibitor (AGA) decreased the PE and CA in secondary bystander A549 cells at all radiation doses under hypoxic conditions. The results support the notion that GJIC could be a possible factor for RISBE under hypoxic conditions. 

Further experiments (Figure 5C,D) showed that when hypoxic-treated bystander A549 cells were incubated with NO inhibitor (c-PTIO) prior to proton microbeam irradiation, the PE and CA of secondary bystander A549 cells were reduced at all doses, which are consistent with the situation of treatment with a GJIC inhibitor. Our results also suggest that NO influences the RIBSE under hypoxic conditions. Overall, these findings support the hypothesis that intercellular communication via GJIC and NO played a key role in the propagation of RISBE-induced damaging effects in secondary bystander cells under hypoxic conditions.

## 4. Discussion 

In RT, lung cancer cells in a state of hypoxia are the most common feature that is indicative of resistance to RT and results in poor clinical outcomes [1,13]. High-LET radiation is thought to be able to overcome hypoxia radioresistance and to improve the therapeutic gain of RT since DNA damage is lessened or does not depend on the oxygen effect. In other words, the biological effects of high-LET radiation were not affected by the oxygen status [14,15]. It is widely known that proton minibeam radiation therapy (pMBRT) is a novel cancer treatment method for reducing damage to healthy normal tissues, but it has an equal effect on tumors as compared to conventional RT [17]. There is evidence that RIBE-induced lethal effects through direct GJIC in both cancer cells and normal cells after exposure to low- and high-LET-radiation [6,9,10,11,12]. Therefore, understanding the mechanism(s) underlying RIBE is particularly important to identify the pathways of cell killing effect. To answer this question, SPICE-QST proton microbeam is the most suitable to determine RIBE in human cells under hypoxic conditions. 

In our previous study, we found RIBE-induced adaptive responses in lung cancer A549 cells under hypoxia but not in normal WI-38 lung cells after high-LET proton microbeams [3]. However, the impact of radiation doses on RIBE under hypoxia remains incompletely defined. Herein, the present results show that hypoxic-treated bystander A549 cells were significantly (* *p* < 0.05 and ** *p* < 0.01) resistant to proton irradiation at all doses by increasing the induction of MN formation in comparison to those in the normoxic group. These indicate that the radioresistance of A549 cells under hypoxia was enhanced (Figure 2B). Our results are consistent with previous studies that showed hypoxia plays a significant role in RIBE-induced radioresistance [18,19,20]. In comparison to normal bystander WI-38 cells under normoxia (Figure 2D), hypoxic-treated bystander WI-38 cells were significantly (* *p* < 0.05 and ** *p* < 0.01) less affected in radioresistant at all doses tested, which was possibly due to the fact that normal cells adapt to chronic hypoxia and, therefore, increase its radiosensitivity characteristics [21,22]. Based on this study, RIBE is seen to depend on cell-type specificity, radiation dose, and oxygen status. 

It has been known that RIBE can possess both radiosensitive and radioresistance properties, depending on GJIC [8]. However, little information is available about the roles of GJIC and radiation doses induced-RIBE in cancer and normal cells after high-LET radiation under hypoxic conditions. Our study showed that hypoxic-treated bystander A549 cells with AGA decreased the RIBE under hypoxia at all doses, resulting in a reduction of cell killing (Figure 3A,B). Conversely, when hypoxic-treated bystander WI-38 cells were treated with AGA, the RIBE was increased at all dose levels (Figure 3C,D). Based on these findings, and in conjunction with our earlier studies, these results support the concept that GJIC-induced RIBE under hypoxia has a different mechanism, both beneficial and detrimental, depending on cell-type specificity [3]. 

RISBE is also important for RT as the primary bystander cancer cells mediate damage or protective signals to their neighboring secondary bystander cancer cells, resulting in the regulation of cell death or survival [7,23]. However, RISBE may affect the therapeutic benefit of RT. Most studies only focused on RISBE under normoxic conditions. Therefore, this study provides the first evidence of the role of RISBE in secondary bystander A549 cells co-cultured with primary bystander A549 cells under hypoxia using a layered tissue co-culture system and irradiated with SPICE-QST proton microbeams (Figure 1). In our experimental conditions, MN formation and CA on truly secondary bystander A549 cells showed a dose-dependent increase when compared to control (Figure 4). Similar to RIBE under hypoxia, RISBE also depends on the radiation dose. More importantly, these results highlighted RIBE- and RISBE-induced damage effects in hypoxic cancer cells (Figure 2, Figure 3 and Figure 4). Therefore, it is important to know the mechanism(s) behind hypoxia-induced RISBE after proton microbeams. These novel findings could be the most important start to understanding tumor hypoxia and increasing the therapeutic potential of RT. 

To better understand the mechanism(s) of RISBE in secondary bystander cells under hypoxia, we further investigated the roles of GJIC and NO in the propagation of toxic effects in secondary bystander A549 cells under hypoxic conditions. Based on our co-culture system, it should be noted that the secondary bystander signals are likely to mediate through GJIC and soluble factors via a cell culture medium. The disruption of GJIC by AGA in secondary bystander cells may be important in understanding the mechanism of radiosensitivity. Our results indicated that GJIC may play a crucial role in the propagation of stressful effects in secondary bystander cells under hypoxia by variant dosage of radiation exposure (Figure 5A,B), possibly due to difference in connexin (Cx) expression [24,25]. Yang et al. [26] have shown that Cx43 expression was increased under hypoxia. Other results suggest that the up-regulation in Cx43 expression is extremely sensitive to radiation [27,28,29,30]. This result implies that Cx43 and other Cx proteins under hypoxia may enhance the radiosensitivity of hypoxic cancer cells. Further research will be required to elucidate the potential Cx proteins for therapeutic development against radioresistance. In addition, these results also suggest that inhibiting GJIC in secondary bystander cancer cells under hypoxia might decrease PE and CA exposure to proton microbeam. Therefore, the restoration of GJIC function in bystander cancer cells by the transfection of CX proteins may play a key role in enhancing cell radiosensitivity and provide a new strategy to overcome the radioresistance of lung cancer.

Furthermore, it has also been reported that NO signaling molecules are involved in RIBE and RISBE under normoxia [8,31,32]. However, less is known about the role of NO and its role in the propagation of RISBE in cancer cells under hypoxia. The treatment of the NO inhibitor (c-PTIO) may decrease MN formation and the CA of secondary bystander A549 cells at all doses tested (Figure 5C,D). This may be attributed to the decrease in DNA double-strand breaks, cellular antioxidants, the accumulation of cyclooxygenase-2 (COX-2) expression, and changes in cytokines in hypoxia-treated bystander cells [7,23,24,33,34]. These findings support the concept that NO plays a role in radiation dose dependence on RISBE under hypoxic conditions. Further studies are warranted to elucidate the molecular mechanisms underlying the role of NO signaling molecules in the propagation of RISBE in hypoxic cancer cells. 

## 5. Conclusions

To our knowledge, this is the first preliminary study to suggest the involvement of intercellular communications via GJIC in the propagation of RIBE- and RISBE-induced stressful effects in hypoxic cancer cells using the SPICE-QST proton microbeams. Specifically, these include the beneficial contribution of RISBE to enhance cancer-killing by radiation, whether through the up-regulation in GJIC or NO production in hypoxic cancer cells during RT. With the advancement of SPICE-QST proton microbeam irradiation technology, these results provide evidence for further in-depth investigation of the mechanism(s) underlying ultra-high dose rate (FLASH)-RT-induced RIBE and RISBE in hypoxic cancer cells and confirm the significance of the role of intercellular communication in the observed effects which may help in improving the treatment of tumor hypoxia.

## Data Availability

Data supporting the findings of this study are available from the corresponding author upon reasonable request.
